# Swarms can be rational

**DOI:** 10.1098/rsta.2024.0136

**Published:** 2025-01-30

**Authors:** Gal A. Kaminka

**Affiliations:** ^1^ Department of Computer Science & Gonda Brain Science Center & BINA Nano-Technology Center Bar Ilan University, Bar Ilan University, Israel

**Keywords:** swarms, rationality, game theory, multi-robot systems, distributed intelligence, collective intelligence

## Abstract

The emergence of collective order in swarms from local, myopic interactions of their individual members is of interest to biology, sociology, psychology, computer science, robotics, physics and economics. *Cooperative swarms*, whose members unknowingly work towards a common goal, are particularly perplexing: members sometimes take individual actions that maximize collective utility, at the expense of their own. This seems to contradict expectations of individual rationality. Moreover, members choose these actions without knowing their effect on the collective utility. I examine this puzzle through game theory, machine learning and robots. I show that in some settings, the *collective utility* can be transformed into *individual rewards* that can be measured locally: when interacting, members individually choose actions that receive a reward based on how quickly the interaction was resolved, how much individual work time is gained and the approximate effect on others. This internally measurable reward is individually and independently maximized by learning. This results in a equilibrium, where the learned response of each individual maximizes both its individual reward and the collective utility, i.e. both the swarm and the individuals are rational.

This article is part of the theme issue ‘The road forward with swarm systems’.

## 1. The perplexing nature of cooperative swarms

Individual members of collectives must interact to achieve their goals, yet their *interaction capacity* is bounded. Individual interaction bounds arise from logical and physical limits on perception range, latency and bandwidth, from analogous limits on the reach and impact of actions and from computational limits on processing incoming and outgoing signals.

By nature, individual interaction bounds do not change with the size of the collective, or its inherent requirements for interaction complexity. When a collective is small, or its inherent interaction requirements are sufficiently low, individuals have no problem interacting with others as needed. However, as collectives grow in size or interactions grow in complexity, individuals are prohibited, by their bounds, from keeping up. Discord and disorder ensue. These may be alleviated when individuals interact explicitly to *organize* the collective, forming *hierarchies* (management, command) and *divisions* (ministries, departments). Otherwise, however, collectives may become *swarms*.

For the purposes of this paper, I define *swarms* intuitively, as collectives of agents whose individual interaction capabilities permit them to interact with a few others (local interactions), but not with all (global interactions). They are flat organizations (no hierarchy), whose members are essentially anonymous and replaceable. Swarms permeate our natural, societal and artificial surroundings. They are found in the collective motion of animals [1–3], human pedestrians [4–6] and traffic [7,8] and multi-robot applications [9–17]. They inspire novel methods for optimization [18,19], safe collision-avoidance [20–24] and future medicinal molecular robots [25–29].

Swarms are investigated not (only) for their ubiquity but for their puzzling nature: they are fantastic exemplars of an ordered system-wide phenomenon arising out of synergistic local interactions between components. Swarm members cannot possibly know the collective state of the swarm, nor can they take actions that directly impact the collective state. Yet time and again, despite the limitations of their constituent members, we see examples of swarms achieving and maintaining an ordered (coordinated) collective state.

*Cooperative swarms*, whose behaviour is understood in terms of collective objectives, are particularly perplexing. Given a measure of collective utility, one could describe a cooperative swarm by its *collective rationality*, the pursuit of the swarm’s members, in aggregate, to maximize the expected collective utility. This follows the definition of the *principle of rationality* [30–33] at the swarm level [34]. For example, the order of a swarm’s collective motion can serve as a measure of collective utility, as could the total amount of food gathered by a foraging swarm, or the rate at which the food is gathered, or the number of items found as swarm members carry out a collective search. Cooperative swarms maximize these measures.

However, introducing rationality as a methodological lens raises significant difficulties when it comes to individuals. While the collective objectives of the swarm may be understood and formalized, their decomposition to individual, self-interested, rational decision making is not at all clear. Agents in cooperative swarms may sometimes need to take actions at their own resource expense, to benefit the swarm. This seems to contradict expectations of *individual rationality*, whereby agents maximize their individually perceived reward. Moreover, even assuming that agents simply adopt the swarm’s collective utility as their own (thus they benefit with others from their individual expenses), swarm agents cannot possibly perceive (measure) the collective utility, as they are limited to local perception. They cannot perceive the collective effects of their actions, and cannot verify that their actions improve the collective utility.

For instance, suppose the collective utility is measured by the total number of food items brought to the nest by foragers. An individual agent can (at best) measure this only when it is at the nest. As it forages, it cannot use the number of food items as a guide to its decision making. Moreover, suppose an agent leaving the nest to forage is about to collide with an incoming forager. Just as it is likely better to let people off the elevator before attempting to move in, the incoming forager may need to back off; but this depends on whether it is holding a food item, how close it is to depositing it and what others (occluded or far) are doing. In swarms, the individuals do not know this information.

Given this difficulty in accounting for individual rationality in cooperative swarms, *Game Theory*, one of the fundamental principled tools for studying multi-agent interactions, is often ignored in cooperative swarm research (see [35,36] for exceptions). Instead, existing approaches rely on task-specific black-box procedures that mechanistically proscribe individual behaviour that increases collective rewards [16], without regarding individual costs and gains. Examples include procedures for collective motion [37–42], area coverage [43–46] and foraging [47–50]. These bypass the question of individual self-interest, and are understood and studied in terms of the collective phenomenon [38,51–58]. They do not lend themselves to analysis from a game-theoretic point of view.

In this article, I present *rational swarms*, a game-theoretic model of *cooperative robot swarms*, as infinite-horizon fully cooperative Markov games (also known as Markov team games), with significant restrictions on the knowledge of the agents. Agents in such games are awarded the collective utility resulting from the joint actions, and thus have an individual incentive to maximize the collective utility. Given their limitations, swarm agents can only receive local rewards, partial proxies of the collective utility. As a result, when they seek to maximize their own local rewards, they can cause the collective utility to decrease, a phenomenon generally referred to as *the price of anarchy*, made famous in the game of Prisoner’s Dilemma.

Focusing on robot swarms, I begin by showing how under modest assumptions, collective utility can be approximated by *aggregating the individual work times of the robots in the swarm*: the times in which they are engaged in their individual tasks, rather than the overhead of coordination. As embodied agents (animals, robots) can be assumed to measure time, this allows swarm members to all use a common, always-accessible, measure of utility. It only requires them to differentiate time and resources spent on their task, from those spent on coordination.

Naively, if each agent maximizes its operational time, and minimizes interference due to miscoordination (e.g. collisions), then the aggregated times increase, and so does the abstract swarm utility [51,56]. However, as the agents know nothing about the effects of their actions on others, attempts to increase their own working time may actually hurt others' efforts.

To address this, I next show that it is possible to reduce collective rewards to individual rewards that are *aligned*, by approximating the individual *difference rewards* [36,59,60]. These turn the Markov game into a potential game [61], where maximizing the individual rewards maximizes the collective rewards [35,62].

**Figure 1.**
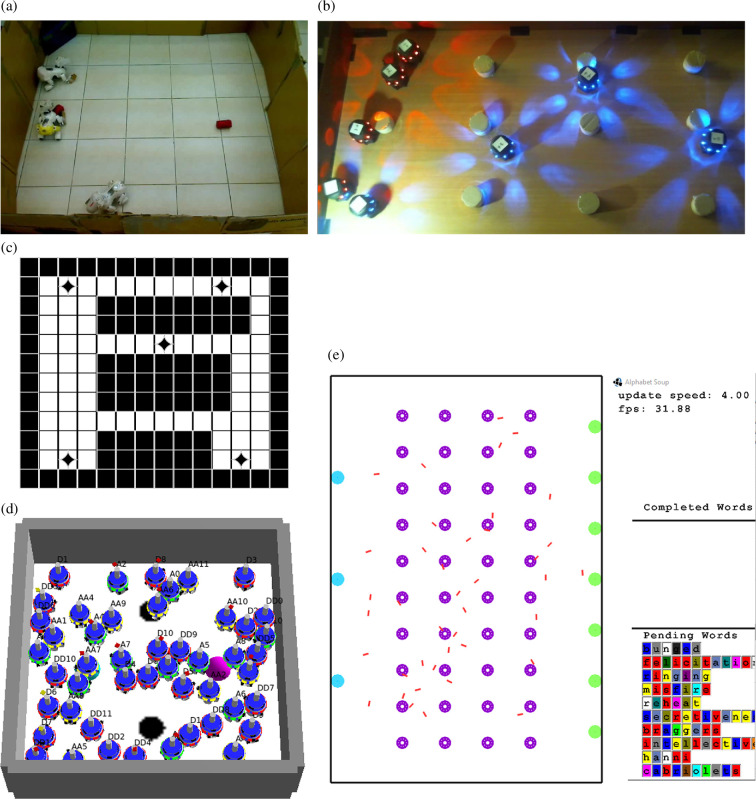
Agents and robots in environments used in experiments with the *rational swarms* model described herein; the agents used reinforcement learning. (*a, b*) Real robots; (*c*) agents moving on a discrete grid; (*d, e*) simulated robots moving in continuous spaces. In all, agents sensed and acted locally, with no communications with their neighbours. Learning was only applied in selecting actions for collision handling. (a) AIBO robots collecting cans [68]. (b) Krembot robots searching for objects [69]. (c) Foraging on a grid [70]. (d) ARGoS [71]-simulated Krembot robot swarms in a search task [72,73]. (e) Alphabet Soup [74] simulation of robots in a warehouse [69].

Using the rational swarms difference reward with distributed multi-agent reinforcement learning [63–67], the robots learn to coordinate and resolve collisions efficiently, maximizing the swarm’s collective utility. The technique has been demonstrated in extensive experiments, in various tasks and settings (figure 1), over the last 15 years.

This article presents a synthesis of the rational swarms model. It begins by showing how the cooperative swarm can be represented as Markov team games, and how the inherent locality of perception of swarm agents can prevent them from individually acting rationally (§2). Then, the rational swarms model is introduced in §3. The §4 discusses lessons learned and insights gained from 15 years of research and development of the model. Finally, §5 discusses the way forward for rational swarms research, and concludes.

## 2. Cooperative swarms as Markov team games

**Preliminaries.** We consider swarms composed of set N of embodied agents, that perceive and act locally, i.e. within some bounded range of their position. The swarm is active for a duration T, which is unknown to us. During this time, each agent i∈N takes actions $a_{t}^{i}$ (action $a^{i}$ taken at time t<T), drawn from a set of possible actions $A^{i}$ by the *strategy* $\pi^{i}$, which determines the action taken by agent i at any given time. Given the local perception of the agents, each individual agent i∈N knows only the action it has taken $a_{t}^{i}$; the effects of the action are localized, affecting only n≪|N| agents (their *social neighbourhood*).

We follow up on previous work [68,69] in distinguishing actions taken by each individual carrying out its task independently of others, and actions taken by agents to coordinate with each other. The need for coordination in swarms arises in two distinct situations. First, agents may inadvertently interfere with each other, i.e. they are *in conflict* [23,51,52,56,68]. This happens for example when their motion trajectories intersect and they (are about to) collide. A second form of coordination may be needed materially for the task, when robots cannot perform a component of the task independently of each other, i.e. more than a single robot is needed to carry out an atomic component of a task (e.g. lifting a long table from both ends requires two robots).

**Figure 2.**
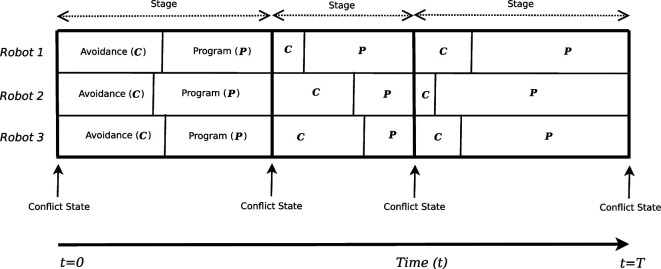
A visual illustration of a swarm member's activity timeline. Duration of *Avoidance* and *Program* modes are implicitly shown by the length of the respective boxes along the horizontal axis. Each stage begins when a conflict state is detected, sending all agents to switch to avoidance mode. Switching back to program mode is done when the conflict is deemed resolved, by each individual separately. Conflict states are assumed to be recognized by all agents.

We focus on swarm tasks where coordination is used to resolve conflicts. In these, the individual agent repeatedly switches between two abstract modes of operation, back and forth: an individual *task*-execution mode (called *Program* mode) where each individual agent is independently carrying out its task within the swarm, and an *Avoidance* mode, where it takes coordination actions to resolve conflicts with other agents, e.g. avoiding collisions. The total operating duration of a swarm member is segmented into *stages*, by conflicts requiring coordination to resolve. Each stage starts with a conflict. Then, coordination actions are taken in avoidance mode to resolve the conflict. This allows the agent to go back to work in program mode, until a new conflict occurs (see figure 2 for illustration).

Multi-robot foraging [49,50,56,75] is a good example. Here, the robots leave the nest in search for food items, which they individually bring back to the nest. Their individual program mode would consist of actions that do not require coordination with others: searching for items, picking them up, finding the way home, dropping collected items. In the course of these activities, they inevitably come near each other (e.g. when some agents are heading with items into the nest area, while others are heading away). Impending collisions trigger a conflict state, which switches the robots to coordination mode, where they take actions to resolve or prevent the collision. Once done, they switch back to program mode.

For now, let us assume that all agents are involved in every collision, i.e. the swarm works from one stage to the next. While all swarm members enter a collision together, they do not necessarily resolve it at the same time: different joint actions may result in some agents leaving the avoidance mode sooner than others. For example, this can occur when three agents collide, and two remain entangled for a while longer, while the third turns away immediately (see figure 2, in the second stage: Robot 1 resolved the conflict earlier than Robots 2 and 3).

We distinguish and focus on cooperative swarms, where a *collective objective* is defined, that agents collectively try to achieve. For example, in collective foraging, the goal of the swarm is to maximize the total number of items collected from the work area. In common collective-motion models, the goal is to establish a common movement heading for the agents. In collective search, the goal is to maximize the number of items discovered. Agents in cooperative swarms individually select actions that maximize achievement of the swarm goals.

This view of the robot’s timeline allows us to position our work with respect to others. Many methods focus on improving the efficiency of the program-mode actions (see e.g. [47,76,77] in foraging). Others focus on improving the entire stage (avoidance and program), by restricting the behaviour of the robot during both program and avoidance, such that collisions and conflicts are minimized [51], e.g. by pre-allocating robots to different areas [78] or tasks [79]. We go beyond the single-agent stage, by examining the stages of all agents, collectively.

**Team (Fully Cooperative) Markov Games.** Each stage (collision resolution and subsequent task activity; figure 2, top) can be viewed as a *normal-form team game*, where the agents all receive an identical payoff for their joint actions. During the avoidance-mode interval of the conflict, agents (*players*) individually select conflict-resolution actions, synthesizing joint actions. In team games, *all agents receive this identical payoff for their joint actions*. Different joint actions can yield different joint rewards, but whatever the joint reward, every agent individually receives it.

The sequence of stages—*subgames*—forms a view of the swarm’s agents as engaged in a *stochastic team game* (also called a Markov team game) [80–83]. A Markov team game is defined by a tuple G:=⟨N,S,A,D,R⟩, where N is the set of agents, S is the set of states (each representing a subgame type, associated with a conflict that could begin a stage), A the set of potential conflict-resolution *joint* actions, D a state-transition probability function and R a function determining the swarm reward from taking an action at a state S. The components of G are explained in detail below. The swarm seeks to maximize the accumulated reward R over many stages. As their number is unknown, this is formally an *infinite-horizon* game [81,82].

**States .** Conflicts are events that interrupt an individual agent’s program mode, and require coordination to resolve. S is the set of recognizable types of conflicts. Collisions are a good example: we can define all collisions to be the same (i.e. a single type, as done in [68,69]), or we can distinguish different types of collisions, especially when the distinction matters to the actions taken. For example, a collision front-to-front may require a different response to a collision front-to-rear, and so the relative position of agents may distinguish conflict states [72]. Similarly, the position of agents in the work area [70], or the local density [84], can also distinguish states.

**The joint actions set A.** The set of all possible joint actions is defined by the sets of potential individual actions $A=A^{1}\times A^{2}\times \cdots \times A^{|N|}$. Each agent i selects its own action $a^{i}\in A^{i}$. Then, a specific joint action a∈A is a tuple $\left(a^{1},a^{2},\ldots ,a^{|N|}\right)$.

**State-Transition Probability Function** D**.** A joint action $a_{t}\in A$ can be applied in any state s∈S. It transitions the swarm from the state s to the next state $s^{\prime }\in S$. This transition corresponds to a single stage (figure 2): the agents begin in a conflict of type s, individually apply $\pi^{i}$ to select avoidance action $a^{i}\in A^{i}$. The individual actions of all agents compose a joint action a, which resolves the conflict and allows each agent to continue in program mode (drawing actions from $\pi_{P}^{i}$). The next state $s^{\prime }$ is determined stochastically, by the transition probability function D:S×A×S→[0,1], measuring the probability of transitioning from state s to state $s^{\prime }$ after applying the joint action a.

**The Collective Reward R.** In the common definition of stochastic games, there are |N|**individual** reward functions $\{R^{i}\},\forall i\in N$, where function $R^{i}$ returns the player’s individual payoff, having been in state s, where the joint action a was taken, and reaching state $s^{\prime }$, determined by the state-transition probability D.

The definition we use here, of team games, differs from the common definition. Instead, we use a single collective reward, R:S×A×S→ℝ, describing *the collective payoff* from performing the joint action a at state s (resolving the conflict), and reaching a new state $s^{\prime }$. All agents share the payoff $R(s,a,s^{\prime })$, as this is a fully cooperative (team) game. Note that R is *stationary*; it does not vary with time, but only with state and action.

**Maximizing the Swarm Collective Reward Accumulated Over Time.** Given a conflict state s, every agent i∈N selects its conflict resolution action $a^{i}$ using its strategy $\pi^{i}$, i.e. $a^{i}:=\pi^{i}(s)$. The individual actions of all agents compose the joint action a that is then applied to the conflict state s to resolve it. This allows the agents to go back to their independent individual programs, until the next conflict state occurs (determined stochastically by D), and a new stage begins. The *joint strategy* of the swarm is defined by the agents’ $\pi :=\left(\pi^{1},\ldots ,\pi^{|N|}\right)$.

We use $s_{t}$ to denote the state of conflict that had begun at time t. At that time, a joint action $a_{t}$ is taken, whose durative effects last until a new conflict arises at time $s_{t^{\prime }}$. The associated stage is denoted $(s_{t},\pi (s_{t}),s_{t^{\prime }})=\left(s_{t},a_{t},s_{t^{\prime }}\right)$. The state $s_{t^{\prime }}$ is a stochastic result of applying the action $a_{t}$; the probability of reaching $s_{t^{\prime }}$ is given by $D(s_{t},\pi (s_{t}),s_{t^{\prime }})$, and the reward for this stage is given by $R(s_{t},\pi (s_{t}),s_{t^{\prime }})$.

A *possible play* η(π) is a sequence $\left\langle (s_{t_{0}},a_{t_{0}}),(s_{t_{1}},a_{t_{1}}),\ldots ,(s_{t_{k}},a_{t_{k}}),\ldots \right\rangle$, generated by repeatedly applying a strategy π to states, beginning with state $s^{0}$ (i.e. $t_{0}=0$), such that for any k∈ℕ, $t_{k}$ is a time-stamp, and $D(s_{t_{k-1}},a_{t_{k-1}},s_{t_{k}})>0$. The accumulating reward over K stages of a *specific* play $\eta_{0}$ is $R_{K}(\eta_{0}):=\sum_{k=1}^{K}R(s_{t_{k-1}},a_{t_{k-1}},s_{t_{k}})$, where $t_{k}$ are specified by $\eta_{0}$.

As the new state $s_{t_{k}}$ resulting from applying $a_{t_{k-1}}$ is determined stochastically by D, a better characterization of the rewards of π is by the *expected* collective reward over K stages, i.e. all of the possible plays π may induce, with K stages:


(2.1) $$\begin{array}{l} \begin{array}{rl} R_{K}(\pi ): & =\;E[R_{K}(\eta (\pi ))] \end{array} \end{array}\;$$

(2.2) $$\begin{array}{l} \;=\sum_{k=1}^{K}\sum_{s_{t_{k}}\in S}[R(s_{t_{k-1}},\pi (s_{t_{k-1}}),s_{t_{k}})\cdot D(s_{t_{k-1}},\pi (s_{t_{k-1}}),s_{t_{k}})]. \end{array}$$

As a notational shortcut, we used $R_{k}(\pi )$ for $R(s_{t_{k-1}},a_{t_{k-1}},s_{t_{k}}),$ and analogously, $D_{k}(\pi )$ for $D(s_{t_{k-1}},\pi (s_{t_{k-1}}),s_{t_{k}})$. Therefore,


(2.3) $$\begin{array}{ll} = & \sum_{k=1}^{K}\sum_{s_{t_{k}}\in S}\left[R_{k}(\pi )\cdot D_{k}(\pi )\right]. \end{array}\;$$

We seek a strategy $\pi^{*}$ that maximizes the expected collective reward generated, in comparison with alternative strategies. However, as K tends towards infinity, as long as R is positive, $R_{K}(\pi )$ will grow unbounded for *any* strategy π. In other words, when K tends to infinity, strategies might not be differentiated by their collective rewards.

We therefore use a standard alternative objective, where the swarm seeks to maximize the *average collective rewards* (limit of means) of $R_{K}$:


(2.4) $$\begin{array}{rl} R(\pi ):= & \lim_{K\to \infty }\frac{1}{K}R_{K}(\pi )=\lim_{K\to \infty }\frac{1}{K}\sum_{k=1}^{K}\sum_{s_{t_{k}}\in S}\left[R_{k}(\pi )\cdot D_{k}(\pi )\right]. \end{array}$$

A strategy $\pi^{*}$ is then one that maximizes R(π):


$$\pi^{*}:=\underset{\pi }{arg\;max}\;R(\pi ).$$

If all the agents know how R changes as a result of their individual actions (i.e. they all receive R), it is straightforward to show (i) that their individual self-interested selections will lead to a Nash equilibrium (i.e. the strategy guarantees stability), and (ii) that the Nash equilibrium also maximizes R (i.e. it guarantees collective reward optimality). Allowing the agents to be rational and self-interested in maximizing their rewards will necessarily maximize the collective rewards, as they are one and the same.

The rewards of the collective are said to be *aligned* with those of the individual [36,60]. In this case, by utilizing reinforcement learning, the agents may evaluate different strategies π by the collective rewards R(π), until converging to the optimal strategy $\pi^{*}$.

**Locality of Perception Leads to Loss of Collective Information.** Unfortunately, the condition that all agents know R does not hold in swarms: the agents cannot measure the collective reward R, as they can only perceive in their immediate local environment. Having no ability to measure R, they naturally also have no way to learn or predict how any joint action, affects it. Individually, they may follow their own self-interested, rational decision-making preferring actions that appear best *from their perspective*, but we lose all guarantees that this will maximize the collective reward.

It may therefore appear that framing cooperative swarms as fully cooperative games yields little insight as to how individual agents should select their actions to maximize the collective reward. As I show next, this is not the case.

## 3. Rational swarms: overcoming locality of perception

The key difficulty with the view of cooperative swarms as Markov team games lies in the fact that swarm agents cannot be assumed to measure the collective reward R, as they can only perceive in their immediate local environment. Thus the first step we take (§3(a)) is to transform the arbitrary collective reward measure into a proxy measure that is individually accessible to all agents. In §3(b) we then show how agents can—in principle—maximize their individually accessible reward, such that it maximizes the collective reward R.

### (a) Local measurement of swarm utility measures

The reward of a cooperative swarm depends on its task, and so is measured in terms that, in general, agents cannot measure directly. While we may model the swarm as seeking to maximize the *average* swarm utility over an infinite horizon (equation 2.4), no agent can realistically measure it. A foraging agent away from the nest cannot keep track of the number of items collected, nor can they assess whether resolving a collision using a specific action is better (improves the R) than another. We therefore seek to transform the utility measure into a form that is accessible to every agent, regardless of where it is.

We focus on *time* as a proxy to the utility gained by swarm members. In particular, we consider the relation between time spent by the swarm members on the task, and the utility resulting from it; we expect maximization of the former to be equivalent to maximization of the latter. As the measurement of time can be carried out individually, without even knowing what the task is, the swarm agents can focus on increasing the time spent on the task, rather than maximizing some abstract notion of utility (this is not trivial to do, see next section).

We remind the reader that we view the swarm’s operation as a series of stages, wherein each agent has two modes of operation: an *avoidance mode*, triggered by collisions or other interference requiring coordination (for which they choose a joint action), and a resulting *program mode* where they carry out their individual tasks, undisturbed.

Here, we examine the agents’ individual intervals of avoidance and program execution, whose duration depends on the individual action taken by each agent (figure 2). The duration of the avoidance interval for agent i∈N is denoted $C_{k}^{i}(\pi^{i})=C^{i}(s_{t_{k-1}},\pi^{i}(s_{t_{k-1}}),s_{t_{k}})$, and the duration of the subsequent program interval is denoted $P_{k}^{i}(\pi^{i})=P^{i}(s_{t_{k-1}},\pi^{i}(s_{t_{k-1}}),s_{t_{k}})$. Their sum is the total duration of the stage $\left[C_{k}^{i}(\pi^{i})+P_{k}^{i}(\pi^{i})\right]=t_{k}-t_{k-1}$. While $C^{i}$ and $P^{i}$ generally vary between agents, their total is common to all agents (as we assume all agents are involved in every conflict). For any stage k, the following holds: $\forall i,j\in N\left[C_{k}^{i}(\pi^{i})+P_{k}^{i}(\pi^{i})\right]=\left[C_{k}^{j}(\pi^{j})+P_{k}^{j}(\pi^{j})\right]$.

It is reasonable to assume that the collective reward grows as agents spend more time in *program mode*, and less in *avoidance* mode, i.e. that it monotonically grows with the collective time spent in program mode. For simplicity, we assume proportionality; there exist constants $\beta^{i},\alpha^{i}\in \mathbb{R}$ where $\beta^{i}>0,\alpha^{i}\geq 0$, such that for any stage $\left(s_{t_{k-1}},\pi (s_{t_{k-1}}),s_{t_{k}}\right)$,


(3.1) $$\begin{array}{r} R_{k}(\pi )=\sum_{i\in N}\left[\beta^{i}P_{k}^{i}(\pi^{i})-\alpha^{i}C_{k}^{i}(\pi^{i})\right]. \end{array}$$

From this we derive the expected time-based reward $\Gamma_{K}(\pi )$ of a policy π over K stages from $R_{K}(\pi )$:


(3.2) $$\begin{array}{lll} R_{K}(\pi )= & \sum_{k=1}^{K}\sum_{s_{t_{k}}\in S}\left[R_{k}(\pi )\cdot D_{k}(\pi )\right] & \text{from equation}\;(2.3) \end{array}\;$$

(3.3) $$\begin{array}{lll} \;= & \sum_{k=1}^{K}\sum_{s_{t_{k}}\in S}\sum_{i\in N}\left[\left[\beta^{i}P_{k}^{i}(\pi^{i})-\alpha^{i}C_{k}^{i}(\pi^{i})\right]\cdot D_{k}(\pi )\right] & \text{from equation}\;(3.1) \end{array}$$

and since D is defined for the joint action $\pi (s_{t_{k-1}})$, it is the same for all agents i∈N,


(3.4) $$\begin{array}{ll} = & \sum_{k=1}^{K}\sum_{s_{t_{k}}\in S}D_{k}(\pi )\sum_{i\in N}\left[\beta^{i}P_{k}^{i}(\pi^{i})-\alpha^{i}C_{k}^{i}(\pi^{i})\right]. \end{array}\;\;$$

(3.5) $$\begin{array}{l} =:\Gamma_{K}(\pi ). \end{array}\;\;\;\;\;\;$$

It is tempting to then redefine R(π) as the limit of $\frac{1}{K}\Gamma_{K}(\pi )$, as K→∞. However, this is incorrect, as it averages over the number of conflicts, giving equal weight to short and long intervals, regardless of their accumulating contributions to the reward. Instead, we should use the average over the accumulating time spent by the swarm agents working through the K stages. The accumulated time *T* is $t_{k}-t_{o}$ (absolute time of the current conflict, minus the absolute time of the initial conflict that marks the beginning of the swarm task). Simply $T=t_{k}$, if we measure the beginning of the swarm task as $t_{0}=0$. However, the conflict state $s_{t_{k}}$ and its time $t_{k}$ are determined stochastically by *D*. Therefore, the total duration *T* varies, depending on π and *D*.


(3.6) $$\begin{array}{lll} \Gamma (\pi ):= & \lim_{K\to \infty }\sum_{k=1}^{K}\sum_{s_{t_{k}}\in S}D_{k}(\pi )\sum_{i\in N}\frac{\beta^{i}P_{k}^{i}(\pi^{i})-\alpha^{i}C_{k}^{i}(\pi^{i})}{t_{k}-t_{0}}. & \text{Plugging in equation}\;(3.5) \end{array}\;$$

(3.7) $$\begin{array}{lll} \;= & \lim_{K\to \infty }\sum_{k=1}^{K}\sum_{s_{t_{k}}\in S}\frac{D_{k}(\pi )}{T}\sum_{i\in N}\left[\beta^{i}P_{k}^{i}(\pi^{i})-\alpha^{i}C_{k}^{i}(\pi^{i})\right]. & T=t_{k}-t_{0}\text{ is same for any}\;i,j\in N \end{array}$$

We now have a measure of the collective reward, which is accessible in principle to any agent i∈N, since it relies solely on *time*, which any of the agents can measure. Noting that the term $\lim_{K\to \infty }\;\sum_{k=1}^{K}\sum_{s_{t_{k}}\in S}\frac{D(s_{t_{k-1}},\pi (s_{t_{k-1}}),s_{t_{k}})}{T}$ is common to all agents, we denote by $\Gamma_{k}^{i}(\pi )$ the contribution of agent i∈N to Γ(π) in stage k−1:


(3.8) $$\begin{array}{rl} \Gamma_{k}^{i}(\pi^{i}):= & \left[\beta^{i}P_{k}^{i}(\pi^{i})-\alpha^{i}C_{k}^{i}(\pi^{i})\right], \end{array}$$

i.e. $\Gamma (\pi )=\lim_{K\to \infty }\;\sum_{k=1}^{K}\sum_{s_{t_{k}}\in S}\frac{D_{k}(\pi )}{T}\sum_{i\in N}\Gamma_{k}^{i}(\pi )$.

$\Gamma_{k}^{i}(\pi )$ (equation 3.8) can be independently computed by agents, and is therefore a step towards compensating for missing information: with every collision, every agent, independently of others, can record the time it spent resolving the previous conflict $C_{k}$ and the time spent working in program mode since the conflict was resolved $P_{k}^{i}$. The terms $\alpha^{i},\beta^{i}$ are also knowable to the agent i. In addition, it also knows T, which is the total time the swarm has been operating, and is therefore the same for all agents.

However, some information remains beyond the immediate perception of the agent, preventing it from computing the collective reward (equation 3.7) from the individual $\Gamma_{k}^{i}(\pi )$ (equation 3.8). In particular, the local perception of each swarm agent i prevents it from knowing $P^{j},C^{j}$ for any agent j≠i∈N. It cannot perceive N, and so does not know how many other agents there are. As a result, agents seem to have little choice but to attempt to rationally maximize their own perceived individual reward $\Gamma_{\pi |i}$, based on their own local knowledge, substituting $\pi^{i}$ for the strategy π, their own locally perceived state $s^{i}$ for s, etc.

As the reward to one agent may be at the expense of another, each agent’s individual reward is no longer *aligned* with the actual collective reward: maximization of one does not lead to maximizing the other. To intuitively see why this happens, imagine some agents are attempting to leave the nest after dropping collected items off, while others are attempting to enter the nest. Those attempting to enter should ideally back off, allowing those inside the nest to go out. However, backing off adds to the duration of the avoidance mode, and reduces the duration of the program mode. Thus those agents are motivated to push forward. This hinders the swarm from collecting items. The next section addresses this challenge.

### (b) Computing a swarm-aligned reward

We can align the individual rewards with those of the collective by finding a *utility potential function* [61]. A utility potential function assigns a scalar (*potential*) to the joint actions of agents, such that any change to the local reward of player i∈N, stemming from its unilateral preference of an individual action $a^{i}$ over a different individual action $a^{i}$, is reflected by a matching change to the potential: if an agent seeks greater individual reward, it will necessarily cause an increase in the potential. Games that possess such a function are called *potential games* [61], and have the property that rational strategic individual preferences for greater local rewards necessarily result in a Nash equilibrium that also maximizes the rewards of others.

Such a potential function was proposed by Wolpert & Tumer [36]. Initially called *Wonderful Life Utility*, it was later renamed and extended as *difference rewards* [59,60], and shown to be related to the economic concept of *marginal contribution* [62,85].

The difference reward Δ(i) of agent i∈N captures the contribution of agent i to the collective reward of the swarm. It is defined as $\Delta^{i}(\pi ):=\delta_{,}^{+i}(\pi )-\delta_{,}^{-i}(\pi )$, where $\delta_{,}^{+i}(\pi )$ denotes the collective reward achieved in the presence (involvement) of the agent, and $\delta_{,}^{-i}(\pi )$ denotes the hypothetical counterfactual reward achieved in the agent’s absence. We discuss this in detail below, and point the reader elsewhere for analytical discussion of the difference reward as a potential [35,62].

#### (i) The collective reward when agent i is present, $\delta_{k}^{+i}(\pi )$.

When agent i∈N is involved (and utilizing strategy π), it is straightforward to take $\delta_{k}^{+i}(\pi )$ to be the collective time-based reward $\Gamma_{k}(\pi )$ at any given stage $k-1=\left(s_{t_{k-1}},\pi (s_{t_{k-1}}),s_{t_{k}}\right)$. We rewrite it using $\Gamma^{i}(\pi )$ to emphasize the role of agent i:


(3.9) $$\delta_{k}^{+i}(\pi ):=\Gamma_{k}(\pi )=\sum_{j\in N}\Gamma_{k}^{j}(\pi )\;\;\;\;\;$$

(3.10) $$\begin{array}{rl} & =\Gamma_{k}^{i}(\pi )+\sum_{j\in N\setminus \{i\}}\Gamma_{k}^{j}(\pi ),\; \end{array}$$

where N∖{i} is the set of agents N, without the agent i. Substituting by the respective definitions, this yields:


(3.11) $$\begin{array}{rl} = & \left[\beta^{i}P_{k}^{i}(\pi^{i})-\alpha^{i}C_{k}^{i}(\pi^{i})+\sum_{j\in N\setminus \{i\}}\left[\beta^{j}P_{k}^{j}(\pi^{j})-\alpha^{j}C_{k}^{j}(\pi^{j})\right]\right]. \end{array}$$

#### (ii) The collective reward when agent i is not present, $\delta_{,}^{-i}(\pi )$.

$\delta_{,}^{-i}(\pi )$ is a counterfactual: it asks what the collective reward would have been, had the agent *not* participated or contributed. Computing the counterfactual takes into account detailed information about the task at hand [60,86], and sometimes can be be computed directly [60]. However, the locality of perception, and thus the limited information available to each agent, makes analytical computation of the counterfactual difficulty. Our one aid in this discussion is that all possibilities are reflective, rather than predictive: any assessment of $\delta_{k}^{-i}(\pi )$ is made at time $t_{k}$, looking back at the duration from the conflict state at time $t_{k-1}$. Thus, the action taken and the resulting duration of the stage $\tau_{k}$ are known at the point of assessment.

We may naively believe $\delta_{,}^{-i}(\pi )$ is equal to $\sum_{j\in N\setminus \{i\}}\Gamma^{j}(\pi )+0$, where the 0 component marks the lack of contribution, of any kind, by agent i (i.e. it is the result of some null action, and $\Gamma^{i}(\pi )=0$). In that case,


$$\begin{array}{rl} \Delta_{k}^{i}(\pi )= & \underset{\delta_{k}^{+i}(\pi ),\;equation\;3.10}{\underbrace{\Gamma_{k}^{i}(\pi )+\sum_{j\in N\setminus \{i\}}\Gamma_{k}^{j}(\pi )}}-\underset{\delta_{k}^{-i}(\pi )}{\underbrace{\sum_{j\in N\setminus \{i\}}\Gamma_{k}^{j}(\pi )-0}}=\Gamma_{k}^{i}(\pi ). \end{array}$$

*However, this is incorrect*. The agents we discuss are *embodied*, necessarily having physical mass and body geometry, and existing over time. When they interact with others, they necessarily affect them. An agent that is not affecting others in a collision is one that simply goes through them, undisturbed and undisturbing. Thus while it is possible for an agent to have zero individual contribution over the duration of a stage ($\Gamma^{i}(\pi )=\beta^{i}P_{k}^{i}-\alpha^{i}C_{k}^{i}=0$), its physical embodied existence affects others during the interval. It is perceived by others, and it can block or facilitate their movement. Furthermore, even if we consider the agent’s hypothetical physical removal from the system altogether, its *absence* would still affect others.

Lacking an analytical model of interactions that is parameterized by information about each individual agent (e.g. its position and velocity, its communicative acts when interacting, etc.), we are left with essentially philosophical approaches for building such a model. We distinguish two components: (i) a model, denoted $\Gamma_{k}^{-j}(\pi )$, of how other agents j≠i are affected when agent i is hypothetically absent from the conflict; and (ii) a model, denoted $\Gamma_{k}^{-i}(\pi )$, for how agent i is affected in this case.

**How others are hypothetically affected.** We argued above that in principle, it is unreasonable to expect all other N−1 agents participating in a collision to be generally unaffected by the absence of agent i. Instead, I consider the direction of the effect.

In principle, the others can do worse without i: their respective avoidance periods grow longer, and necessarily their productivity decreases, i.e. $\Gamma_{k}^{-j}(\pi )<\Gamma_{k}^{j}(\pi )$. In the extreme case, they hypothetically remain in avoidance mode for the duration of the stage, and so,


(3.12) $$\begin{array}{lrr} & \Gamma_{k}^{-j}(\pi ):=-\alpha^{j}\tau_{k}=-\alpha^{j}\left[P_{k}^{j}(\pi^{j})+C_{k}^{j}(\pi^{j})\right]. & \end{array}$$

Adapting this interpretation requires a narcissist view of agent i’s importance to the interaction: without its involvement as a benefactor, others are unable to proceed.

The opposite view is that without agent i, the conflict did not occur (i.e. agent i is a disruptor, the only cause for a conflict). In that case, the other agents would have spent the duration of their now-hypothetical avoidance interval working their own task (program mode), and the avoidance mode duration for all agents j∈N∖{i} would have been as rewarding as the program mode. Instead of reducing the reward by $-\alpha^{j}C_{k}^{j}$, we *increase* the reward by $\beta^{j}C_{k}^{j}$, as the entire duration of stage k is considered to have been spent by any agent j carrying out its program:


(3.13) $$\begin{array}{lrr} & \Gamma_{k}^{-j}(\pi ):=\beta^{j}\tau_{k}=\beta^{j}\left[P_{k}^{j}(\pi^{j})+C_{k}^{j}(\pi^{j})\right]. & \end{array}$$

The two models mark extreme points that bound the true values from above and below. If agent i was responsible for the conflict, its removal from the system increases everyone’s productivity up to $\beta^{j}\left[P_{k}^{j}(\pi^{j})+C_{k}^{j}(\pi^{j})\right]$ (equation 3.13). If instead agent i was the benefactor, whose presence allowed the conflict to be resolved, then everyone’s productivity would be reduced to $-\alpha^{j}\left[P_{k}^{j}(\pi^{j})+C_{k}^{j}(\pi^{j})\right]$ (equation 3.12) in its absence.

**How agent** i **is hypothetically affected.** Agent i knows its own actual $\Gamma_{k}^{i}$. There are several ways of approaching the question of how this value changes when i is absent from the system. Clearly, one possibility is indeed to assume that it in its absence, its own counterfactual contribution is 0 (as discussed above):


(3.14) $$\begin{array}{lrr} & \Gamma_{k}^{-i}:=0\leq \Gamma_{k}^{i}. & \end{array}$$

I see two other interpretations, that bound the unknown real value from above and below. One possibility is that the agent i had spent the entire stage in avoidance mode, i.e. it is absent from the stage in the sense that it is not producing anything:


(3.15) $$\begin{array}{lrr} & \Gamma_{k}^{-i}:=-\alpha^{i}\tau_{k}=-\alpha^{i}\left[P_{k}^{i}(\pi^{i})+C_{k}^{i}(\pi^{i})\right]. & \end{array}$$

The other interpretation is that in its absence from the conflict, it never went through the avoidance interval, and so,


(3.16) $$\begin{array}{lrr} & \Gamma_{k}^{-i}:=\beta^{i}\tau_{k}=\beta^{i}\left[P_{k}^{i}(\pi^{i})+C_{k}^{i}(\pi^{i})\right]. & \end{array}$$

**Putting** $\Gamma_{k}^{-j}(\pi )$ **and** $\Gamma_{k}^{-i}$ **together.** We can now define the counterfactual using the above. While multiple possibilities exist, most recent work [72,73] empirically demonstrated that it is useful to take the so-called *benefactor* view, whereby without agent i, the conflict did not occur (or at least, the avoidance duration decreased [69,87]) (equation 3.13), while the agent’s hypothetical contribution in its absence is 0 (equation 3.14):


(3.17) $$\delta_{k}^{-i}(\pi ):=\Gamma_{k}^{-i}(\pi )+\sum_{j\in N\setminus \{i\}}\Gamma_{k}^{-j}(\pi )\;$$

(3.18) $$\begin{array}{ll} = & \underset{equation\;(3.14)}{\underbrace{0}}+\sum_{j\in N\setminus \{i\}}\underset{equation\;(3.13)}{\underbrace{\left[\beta^{j}P_{k}^{j}(\pi^{j})+\beta^{j}C_{k}^{j}(\pi^{j})\right]}} \end{array}.$$

#### (iii) Putting it all together: an aligned individual reward, $\Delta_{k}^{i}(\pi )$

$\Delta_{k}^{i}(\pi )$ can now be defined as follows:


(3.19) $$\Delta_{k}^{i}(\pi ):=\delta_{k}^{+i}(\pi )-\delta_{k}^{-i}(\pi )\;$$

(3.20) $$\;\begin{array}{ll} = & \overset{\delta_{k}^{+i}(\pi ),\text{equation}\;3.10}{\overbrace{\beta^{i}P_{k}^{i}(\pi^{i})-\alpha^{i}C_{k}^{i}(\pi^{i})+\sum_{j\in N\setminus \{i\}}\left[\beta^{j}P_{k}^{j}(\pi^{j})-\alpha^{j}C_{k}^{j}(\pi^{j})\right]}}-\overset{\delta_{k}^{-i}(\pi ),\text{equation}\;3.18}{\overbrace{\sum_{j\in N\setminus \{i\}}[\beta^{j}P_{k}^{j}(\pi^{j})+\beta^{j}C_{k}^{j}(\pi^{j})]}} \end{array}$$

(3.21) $$\;\begin{array}{ll} = & \beta^{i}P_{k}^{i}(\pi^{i})-\alpha^{i}C_{k}^{i}(\pi^{i})+\sum_{j\in N\setminus \{i\}}\left[\beta^{j}P_{k}^{j}(\pi^{j})-\alpha^{j}C_{k}^{j}(\pi^{j})-\beta^{j}P_{k}^{j}(\pi^{j})-\beta^{j}C_{k}^{j}(\pi^{j})\right] \end{array}\;$$

(3.22) $$\;\begin{array}{ll} = & \beta^{i}P_{k}^{i}(\pi^{i})-\alpha^{i}C_{k}^{i}(\pi^{i})-\sum_{j\in N\setminus \{i\}}(\alpha^{j}+\beta^{j})C_{k}^{j}(\pi^{j}) \end{array}.\;$$

Note that when the agent *i* is alone (no neighbours, i.e. N∖{i}=∅), $\Delta_{k}^{i}(\pi )$ is simply its own time-based reward $\Gamma_{k}^{i}(\pi )=\beta^{i}P_{k}^{i}\left(\pi^{i}\right)-\alpha^{i}C_{k}^{i}\left(\pi^{i}\right)$. The aligned difference reward elegantly simplifies to the natural individual reward in this case. There is no need for the agent to switch between reward functions depending on its social settings.

### (c) Determining $\pi^{*}$ by reinforcement learning

Through a completely distributed learning process, every agent i∈N uses its own computed $\Delta_{k}^{i}$ to learn its own optimal strategy $\pi^{i}$, maximizing


$$\begin{array}{r} \lim_{K\to \infty }\;\sum_{k=1}^{K}\sum_{s_{t_{k}}\in S}\frac{D_{k}(\pi )}{T}\Delta_{k}^{i}(\pi^{i}), \end{array}$$

such that the composed *joint* policy is optimal $\pi^{*}$. While equation (3.22) removes some of the knowledge requirements from the individual agent (e.g. it does not need to know $P^{j}$ and T), it still leaves some unknowns ($N,\alpha^{j},\beta^{j},P^{j},C^{j}$). These will be approximated below. The transition probability function D is not known to the agent, and will be addressed through the learning process.

First, assuming agents are homogeneous, we may set $\alpha^{i}=\alpha^{j},\beta^{i}=\beta^{j}$, and rewrite equation (3.22):


(3.23) $$\begin{array}{ll} \Delta_{k}^{i}(\pi^{i})\approx & \beta^{i}P_{k}^{i}(\pi^{i})-\alpha^{i}C_{k}^{i}(\pi^{i})-\sum_{j\in N\setminus \{i\}}\underset{\text{replacing }\alpha^{j},\beta^{j}}{\underbrace{\left(\alpha^{i}+\beta^{i}\right)}}C_{k}^{j}(\pi^{j}). \end{array}$$

Next, we can estimate the unknown duration $C^{j}$ by the mean duration of $C^{i}$ in previous stages, i.e. $\bar{C_{K}^{i}}(\pi^{i}):=\frac{1}{K}\sum_{k=1}^{K}C_{k}^{i}(\pi^{i})$. Both last assumptions follow a common approach in multi-agent reinforcement learning [88], where the learners assume others are similar. Using this last estimate, we can continue rewriting,


(3.24) $$\begin{array}{lll} \approx & \beta^{i}P_{k}^{i}(\pi^{i})-\alpha^{i}C_{k}^{i}(\pi^{i})-\sum_{j\in N\setminus \{i\}}(\alpha^{i}+\beta^{i})\bar{C_{k}^{i}}(\pi^{i}) & \left(C_{k}^{j}(\pi^{j})\approx \bar{C_{k}^{i}}(\pi^{i})\right) \end{array}$$

and finally, as N∖{i} is unknown, we use the locally perceived social neighbourhood, of size n≪|N| (n here includes the agent i) as a sufficient estimator [72,73,84]. The intuition for this is that in collisions, agents that are not sensed are not part of the collision. They are therefore not affected by it, nor do they affect it, and so their C duration is 0, having no impact on $\Delta_{k}^{i}(\pi )$ (their P duration is not needed for the computation in any case). We therefore finally rewrite


(3.25) $$\begin{array}{lll} \approx & \beta^{i}P_{k}^{i}(\pi^{i})-\alpha^{i}C_{k}^{i}(\pi^{i})-(n-1)(\alpha^{i}+\beta^{i})\bar{C_{k}^{i}}(\pi^{i}) & \text{(local neighbours)}. \end{array}$$

This last step touches on a key assumption in the model, that conflicts involve all agents in N. This assumption is generally violated in swarms, and most certainly when we examine collisions as the source of conflicts: there is no reason, and no possibility, that all robots collide together. The intuition provided above for the use of only the n robots locally perceivable by the robot argued on practical grounds that the C,P can be ignored or nullified. However, this is a practical approximation. Theoretically, the assumption still needs to be addressed.

From a mathematical point of view, Douchan [84] shows that it is enough to consider agents that are not involved in a conflict to have selected a special individual action $a_{P}$, which has a C duration of 0, and therefore is maximally productive (has a program interval of length $\tau_{k}$). However, this mathematical equivalence is not amenable to local approximation. In future work, the rational swarms model should be developed to address *sparse interactions*, by appropriate shaping of the reward [89] or using specialized algorithms [90].

Necessarily, each agent uses its own *estimated* $\Delta_{k}^{i}(\pi^{i})$ (equation 3.25), using no information about others. Agents are therefore *independent learners* in multi-agent reinforcement learning. Despite the well-known inherent challenges of using reinforcement learning with independent learners [63–66,82,91–94], many algorithms have been developed to allow cooperative learning in multi-agent settings [67,81,92,95,96]. Surprisingly, the approach presented here has been repeatedly demonstrated to reach stable and highly optimized collective swarm rewards in many experimental settings, using the most basic of algorithms (see discussion below). Note that as the rational swarm model is stated—naturally—as an average (undiscounted) rewards optimization criterion, rather than discounted rewards, some algorithms may be better suited than others [97–99].

## 4. Lessons from experiments with artificial rational swarms

Over the last 15 years, the rational swarms approach was investigated in different experimental settings, involving embodied agents and robots (figure 1). The discussion above presents an up-to-date perspective, which allows us to view earlier work as special cases and approximations. In this section, I highlight insights from experiments carried out as part of the research. The next section (§5) will discuss future work, informed by this perspective.

Working in continuous spaces, with no communications between the agents, the rational swarms approach used with reinforcement learning (§3) has been consistently demonstrated to achieve superior swarm collective rewards when compared with manually tuned collision-avoidance methods (including methods allowing stochasticity in algorithm selection) [68–70,72,73,87,100,101]. A bird’s eye view of the experiments reveals the importance of both components of the model: the use of time as a measure of utility (§3(a)), as well as the alignment of individual and collective rewards (§3(b)).

Earlier formulations and experiments [68,70] did not account for alignment of collective and individual rewards, instead nullifying the impact on others. They focused on minimizing the impact of the avoidance interval (C), ignoring the program interval and its gains for the most part. Later experiments used various alignment approximations, and produced superior results [69,87], though still focusing on the avoidance interval. These also demonstrated that the aligned time-based rewards worked on-par, or even better, than aligned rewards using the utility measure used in the task (e.g. number of items picked). We believe that this is due to the time-based difference rewards offering a more sensitive, finer-resolution measure; similar observations were also made by others [60,86].

More recently [72,73] the rational swarms model was clarified and simplified, as a result of mathematical derivation of the time-based collective reward from the perspective of the swarm. The model presented in §3 is a generalization and synthesis of these latter investigations. It builds on the empirical evidence to prefer the so-called *benefactor* view, whereby without agent i, the conflict did not occur (or at least, the avoidance duration decreased [69,87]), and also the use of equation (3.14) ($\Gamma_{k}^{-i}=0$) for the agent’s own counterfactual contribution. However, the other models are useful as analytical bounds [87].

The success of the rational swarms model in *continuous* settings is particularly noteworthy. Most of the work on Markov games and much of the literature on game-theory in general is carried out in discrete settings and discrete time (differential games being the notable exception [102,103]). It is therefore not straightforward to be able to apply a model based on Markov games, with its assumptions of discrete actions and states, to continuous settings.

I believe the observed success in continuous action spaces owes much to the use of *collision-avoidance algorithms* as *actions* used by the individual (i.e. composing the set of actions $A^{i}$). Each algorithm works as a *macro-action* or *option* [104]: once selected, it takes over control of the agent, generating motion actions at a fine resolution to resolve the collision. Deciding on a macro-action is carried out at a higher level of abstraction than the fine resolution of continuous motion. Robotics literature reports on many such algorithms [20,21,23,24,105–107]. In fact, this research direction began by observing that no one algorithm could be shown to be superior [23,52]. It seemed reasonable to let the swarm learn *when* and *which* algorithm to use.

More specifically, as agents learn independently of each other, each agent i learns its own strategy $\pi^{i}$ that determines which algorithm to apply, and when. The strategy may differ from that of other agents. A repeating observation is that the swarm *rarely* converged to a homogeneous choice of collision-avoidance algorithms. Rather, in the great majority of experiments, the post-learning swarm was composed of groups, each distinguished by the fact that its member agents had learned to deal with collisions using a specific macro-action, different from those of other groups. In other words, the swarm has learned to diversify, in terms of collision-handling responses. Repeatedly, it had become *behaviourally heterogeneous* [53,108], even if the robots are physically homogeneous (contrast with investigations on heterogeneous robots [109–111]). Kaminka & Douchan [87] present detailed results analysing the behavioural heterogeneity of the swarm, albeit using a dated variant of the rational swarms model.

The use of a limited set of macro-actions also greatly simplifies the requirements from the learning mechanism used. Although there has been much progress in utilizing reinforcement learning in continuous spaces, and especially in robotics [112–116], the model results in simple formulations of the learning settings: so-called *stateless* settings (equivalent to classic multiarm bandits), where every conflict state is considered to be identical to any other (i.e. |S|=1, in which case the Markov game model above is reduced to a *repeating games* model [68,69]); or settings including only a handful of states, distinguished by the local density of the social neighbourhood of the agents [84], the position of the agents [70], or the side of the collision [72]. In these simple settings, even the simplest classic algorithms for reinforcement learning work well in practice: *UCB1* [99] for multiarm bandits, and *Q-Learning* [117,118] for multi-state settings.

Some of the lessons from these 15 years of investigations are negative in nature, and point the way towards needed improvements in the theory and practice of rational swarms. Most importantly, while the use of the rewards is robust (i.e. the agents learn), it is necessary to carefully check that the proportionality assumptions underlying equation (3.1) (relating the rewards to the time spent in program and avoidance modes) are maintained. We had, on occasion, discovered that robots learned to move about doing essentially nothing when the underlying program was ineffective (i.e. $\beta^{i}$ was close to 0). A related challenge for future research is to allow associating actions $\pi^{i}(s)$ with their time constants α or β. This, for instance, would allow us to differentiate collision-avoidance algorithms that take the same amount of time (i.e. their resulting $C^{i}(\pi^{i}))$ is the same), but they have other costs differentiating them (e.g. energy consumption).

Two important challenges are raised in using the rational swarms model with real robots. First, robots may face difficulties distinguishing collisions with walls, from collisions with other robots. Such a distinction is critical: collisions with robots signify conflict, while collisions with walls are handled as part of the robot program or avoidance modes, without changing the conflict state. Second, attempting to introduce states into the learning process (e.g. distinguishing actions taken in different spatial arrangements of neighbours around the robot) results in a combinatorial explosion in the number of states, a problem that is exacerbated when we consider continuous spaces. The use of state-decomposition or neural network methods may be useful.

## 5. The way forward: natural and artificial rational swarms

To the best of my knowledge, the rational swarms model offers the first game-theoretic view of cooperative robot swarms, bridging a perplexing gap between the rationality of the swarm as a collective, and the individual rationality of its members. Continued development of the model is informed through empirical work. The model is currently being investigated as a basis for analysis and development of simulated and real robot swarms carrying out various cooperative tasks. It has already been extended and demonstrated in swarm-competitive foraging [73], where two or more cooperative swarms compete with one another with respect to the number of items collected.

New tasks point the way towards needed generalization and sophistication, that promise to broaden the applicable scope of the approach, and deepen its impact. For example, *collective transport* is a foraging variant, where some items require more than one robot to carry: some items require multiple robots to carry. This simple variation changes the underlying assumption of the model: here, robots coordinate not only to avoid interfering with each other, but also to carry out their task. In terms of the rational swarms approach, this requires extending the approach to address coordination during program mode. Such extensions will also offer an opportunity to interact with orthogonal investigations by others, for improving individual foraging: better search patterns, localization capabilities, homing, etc. [47,49,119].

A primary open question, raised empirically to be addressed theoretically, deals with bounds on errors due to estimations used in the calculation of the difference reward, in particular of the counterfactual collective reward without the participation of the agent. A few steps towards such bounds are discussed in §3(b)-ii: upper and lower bounds for counterfactual values are presented, and may be used in future theoretical analysis.

However, other components in the rational swarms model are also approximated. For instance, the specific agent’s mean experience (mean duration of avoidance) is used as an estimate for the contributions of others. This follows in the general spirit of assumptions, that the agents are homogeneous, anonymous and replaceable. However, it has no formal basis, and an empirical investigation of this estimator versus others (e.g. min⁡ or max⁡) in [84,87] proved largely inconclusive. These issues remain open for future investigations.

Perhaps the most urgent open question is that of the convergence of the aggregated individual difference reward (equation 3.22), or its approximation in equation (3.25), to the time-based collective reward (equation 3.7). To my best knowledge, no theoretical proof exists that the difference reward, when used in a distributed fashion, convergence to the maximum-payoff social–welfare equilibrium in which the collective reward is maximized. Yet empirically, it seems to do just that across a wide variety of environments. The challenge is exacerbated because in independent learners settings (the case here), multi-agent distributed learning is hampered by the non-stationary nature of the rewards, and the potential existence of multiple equilibria [66,81,92,96].

More generally, despite the promise of the rational swarm model in terms of providing an individually rational account of cooperative swarms, I note that its current state offers no predictions at a fine resolution (e.g. the time that it would take for the swarm to reach maximum mean collective reward), nor tools for analysis (e.g. would the swarm end up being heterogeneous in its individual strategies, and to what degree). These are questions that are open for many models of swarms, and restrict both our understanding of natural swarms and our use of synthetic swarms in important application areas. A detailed predictive model of foraging is presented by Lerman & Galstyan [56], demonstrating that such predictions are possible.

The use of robots as exploratory tools for investigating swarms is a direct connection to applications of swarm robotics. It is also an approach to investigating swarms synthetically. Robots and animals are both embodied, and share design constraints: energy use, geometrical and kinematic constraints restricting motions, noise in sensing and actuation, computational processing limitations and more. Understanding of animal swarms can and does inform our understanding of synthetic swarms. However, the reverse can also be true [120–122].

I wish to highlight two example swarm research areas, one of natural swarms, and one of synthetic swarms, to illustrate both the promise of the rational swarms model and the questions it leaves open:

1. *Are Swarm Animals Individually Rational?* The nymbot–locust hybrid swarm [123,124] mixes locust nymphs and robots in laboratory settings. The investigation seeks to answer fundamental questions about individual locust decision making, by using robots to conduct controlled experiments; controlling robot swarm motions, we measure the animal responses. It also attempts to construct algorithmic models of natural behaviour (see [4,6,122], for like-minded modelling attempts).

In principle, the rational swarms model is applicable here (to guide robot motions). However, a key open question touches on its suitability for *modelling the animals*, not *driving the robots*. Is there a detailed account of individual animal decision making, that is both individually rational, as well as collectively optimal? In other words, can we demonstrate that the locust (or other animals) perceive their neighbours through the transformation imposed by a difference reward? Do they use internal time measurements as part of their reward?

2. *Molecular Medicinal Robot Swarms.* There are many investigations of nanometer-scale molecular devices, some as simple as particles whose size and shape yield medically useful results, some as complex three-dimensional structures with local actuation [125]. These so-called *nanobots* offer an opportunity for clinical targeting of specific organs or biosites, which are not typical of more familiar types of medicine. While most investigations focus on the affinity between the device and its target location, there is growing evidence that by combining different nano-devices, i.e. creating a *heterogeneous drug swarm*, better results can be achieved [25–29,126].

These advanced therapies necessarily require consideration of the interactions of nano-devices within the body, by direct chemical reactions [26–28] or through synergistic interactions in the bio-chemical environment [25,29,126]. The extreme limitations of nano-devices inherently mean that they are inherently and myopically 'selfish', following chemical gradients and reactions, with no capacity for prediction or foresight. As medical applications require the devices to serve a collective medical goal, a method is needed to align the greedy, self-interested behaviour of the nano-devices (viewed here as nanobots) with the goals of the swarm. I envision a biochemical version of the rational swarm model—if developed for biochemical use—whereby it is used to plan both the construction and reactions of nanobots such that the affinity of different particles with respect to each other or target areas is guaranteed to achieve a clinical collective result. In other words, the nanobots would be designed such that they follow a collectively aligned gradient.

## 6. Conclusions

The research presented in this paper, and the future directions described above, should be understood in the context of a call to arms, for multidisciplinary research into *rational* swarms, to fill our world in its natural as well as in its urban, technological aspects. Natural and synthetic swarms are similarly constrained and can share analysis and modelling approaches, as have been demonstrated. The focus on rationality of swarms, from a multidisciplinary perspective, can generate significant impact on all disciplines involved in swarm research.

## Data Availability

This article has no additional data.
